# Change in decision-making skills and risk for eating disorders in adolescence: A population-based study

**DOI:** 10.1192/j.eurpsy.2020.92

**Published:** 2020-10-13

**Authors:** M. Francesconi, E. Flouri, A. Harrison

**Affiliations:** Department of Psychology and Human Development, Institute of Education, University College London, London, United Kingdom

**Keywords:** Cambridge gambling task, decision-making, eating disorder, latent class analysis, Millennium Cohort Study

## Abstract

**Background:**

Despite the growing interest in the involvement of decision-making under conditions of risk in the onset of eating disorders in adolescence, no studies have investigated how the development of decision-making across that period may influence such a risk. Using data from the Millennium Cohort Study this study explored whether changes in performance on the Cambridge Gambling Task (CGT) between age 11 and age 14 were associated with presence of eating disorder (ED) symptoms at age 14.

**Methods:**

Latent class analysis was used to identify groups with distinct profiles based on their responses to questions investigating eating and dieting at age 14. CGT change scores were used as predictors of latent class membership in a logistic regression while accounting for confounders.

**Results:**

In our sample of 11,303 participants, the best class solution was a two-class one reflecting high and low risk for ED symptoms. Higher risk-taking scores and lower quality of decision-making scores at age 11 were associated with increased odds of belonging to the high-risk group at age 14. Risk-taking was reduced from age 11 to age 14, but a smaller reduction was associated with a higher probability of being in the higher risk group at age 14. The change over time in the other CGT measures was not associated with risk for ED symptoms.

**Conclusions:**

Atypical change in risk-taking from early to middle adolescence may be implicated in the risk of ED symptoms in middle adolescence. These results should be replicated in clinical samples.

## Introduction

Eating disorders (EDs) are severe debilitating mental illnesses with the highest mortality rate of all mental illnesses [1]. Mortality is the most extreme prognosis but other outcomes associated with EDs include long-term impairments such as poor functioning, unemployment, physical complications, and other mental comorbidities [2,3]. Remission rates in treated samples range from 19 to 65%. The onset of EDs typically occurs during adolescence or early adulthood and a growing body of studies shows an increase in the incidence and prevalence of EDs in children [4,5]. These data emphasize that early identification of modifiable risk factors for the development of EDs is key in achieving better outcomes [1,6,7].

One such factor may be disadvantageous decision-making under conditions of risk [8,9], related to the probability of risky behaviors and a host of adverse life outcomes [10]. An explanation why it may also be related to the risk of EDs is that making inefficient decisions under conditions of risk may reflect impulsivity and anxiety traits known to play an important role in the genesis of dysfunctional behaviors, including EDs [11]. There is indeed evidence that overly cautious responding on tasks measuring decision-making under conditions of risk is associated with restricting EDs, whereas more impulsive responses have been associated with binge-purge EDs [12,13].

Arguably, investigating the role of decision-making under risk in early and middle adolescence for EDs may be especially informative. That period, due to the difference in the pace of maturation of the ventromedial prefrontal cortex compared to that of the dorsolateral prefrontal cortex [10,14], is very important for studying the development of decision-making skills [10,15]. To our knowledge, this role has only been recently investigated in the general population. For example, in our previous (unpublished) study we explored this by measuring decision-making under risky conditions with the Cambridge gambling task [16,17] (CGT). The CGT, used in the present study as well, has been developed to assess decision-making and risk-taking behavior outside a learning context. We found that higher scores in risk-taking and lower scores in quality of decision-making at age 11 were associated with the risk of developing EDs at age 14 (Harrison, Francesconi & Flouri, submitted).

What we have not investigated, and is still unknown, however, is how the *development* of decision-making from early to middle adolescence may be associated with the presence of ED symptoms. This is important especially considering that decision-making is highly sensitive to age-related change, particularly in adolescence [18]. In contrast to the typical linear development of executive functions, for example, affective decision-making abilities progress non-linearly, declining from late childhood to early adolescence, and improving during mid-adolescence [10]. We argue that atypical patterns of change in decision-making skills from early to middle adolescence may be particularly useful for understanding the etiopathogenesis of mental illnesses associated with decision-making difficulties, including EDs. Therefore, this study aimed to investigate the role of change in decision-making skills from early to middle adolescence in the development of ED symptoms in middle adolescence, while taking into account potential confounders, in a large general-population cohort. It was hypothesized that change in decision-making skills, measured using the CGT-derived variables (risk-taking, quality of decision-making, deliberation time, risk adjustment, and delay aversion) between the ages of 11 and 14 in the participants of the Millennium Cohort Study (MCS) would be associated with the probability of belonging to groups that differ in terms of important ED symptoms (body dissatisfaction, intention to lose weight, dietary restriction, significant under/overweight, and driven exercise to influence weight/shape) at age 14.

## Methods

The MCS is a nationally representative longitudinal study of 19,244 infants who were born across England, Scotland, Wales, and Northern Ireland in 2000–2002 [19]. In MCS certain sub-groups of the population were intentionally over-sampled, namely children living in disadvantaged areas, children of ethnic minority backgrounds, and children growing up in the three smaller nations of the UK. The disproportionate representation of these groups ensures that typically hard to reach populations are adequately represented and that sample sizes are sufficient for the analysis of ethnic minorities, those from disadvantaged backgrounds and children within each of the UK nations. There have been six sweeps of data collection to date. In this study, we used data from sweeps 5 (age 11) and 6 (age 14). Our analytic sample included singletons and first-born twins or triplets with available information on eating, dieting, or body image questions at age 14 and with available data on the CGT at age 11 or 14 (*n* = 11,303).

### Measures

The CGT [17] is an experimental subtest of the widely used and well-validated Cambridge Neuropsychological Test Automated Battery [20] that measures *decision-making under risky conditions*. Participants see a row of 10 boxes (red and blue) across the top of the computer screen and are told that a token is hidden behind one of them. The task consists of five stages, each of which is a block of trials. In the first, decision only stage, participants simply have to guess whether the token is hidden under a red or blue box. The latter four stages are gambling stages where, following the color decision, participants bet a proportion of their points (from an initial 100 on each stage) on their confidence in the location of the yellow token. Two of the gambling stages are practice sessions undertaken prior to a test session, so that participants’ performance is ultimately assessed by the two test gambling stages. In the gambling stages, participants start with a number of points displayed on the screen and select a proportion of these points, displayed in either rising or falling order, in a second box on the screen to gamble on their confidence in this judgment. A stake box on the screen displays the current amount of the bet. Participants are informed that correct bets will be added onto their points score (and incorrect ones will be subtracted from it) and that they should try to win as many points as possible.

Participants have to choose (a) which color of box they believe the token is hidden behind (red or blue) and (b) the number of points they want to gamble. The proportion of red to blue boxes (box ratio) varies during the task pseudo-randomly to assess the influence of statistical risk (probability) on decision-making. The five CGT measures of decision-making that are used in this study include: (a) risk-taking, the mean proportion of points bet on trials where the most probable color was selected; (b) quality of decision-making, the mean proportion of trials where the most probable color was selected; (c) deliberation time, the mean time (in milliseconds) taken to make a box color selection; (d) risk adjustment, the extent to which betting behavior is moderated by probability; (e) delay aversion, the time participants are prepared to wait in order to place a higher or lower bet. A sixth CGT measure, overall proportion bet (i.e., the mean proportion of points gambled across all trials) was excluded from our analysis in view of its very high correlation (>0.90) with risk-taking. Further details on the outcome measures from the CGT are given in Table S1 in the supplementary material.

In the MCS, a series of questions were asked at age 14 to assess *eating and dieting attitudes and behavior*, which correspond to key diagnostic criteria provided by the American Psychiatric Association (2013). These dichotomous items (yes/no) measured: body dissatisfaction (one item measuring whether the participant perceived themselves as too overweight or not); intention to lose weight (one item measuring the presence of a strong desire to lose weight); dietary restriction (one item measuring whether the participant had ever actively eaten less to influence their shape/weight) and excessive exercise (one item measuring whether the participant had ever exercised in a driven way in order to influence their weight and shape). In MCS, height, weight, and body fat measurements were taken by the interviewer at age 14 for each cohort member. Therefore we also included in our analysis an objective measure of underweight and overweight based on the most widely used reference panel, the UK90, which is sensitive to gender and age and developed for the British population. Cut-offs in our sample were based on the age of the cohort member at the time of interview. The underweight cut-off point was the second centile and the overweight cut-off point was the 85th centile, as suggested by the UK 90 [21].

We controlled for a number of *covariates* known to be associated with exposure and outcome, including gender, ethnicity (according to the UK census groups of white, black, Indian, Pakistani/Bangladeshi, mixed, or other), family poverty at age 11 (below the poverty line or not), IQ, derived in MCS at age 5 from three subscales of the British Ability Scales (BAS) [22], pubertal status at age 11 (breast growth or menstruation or hair on body for females, and voice change or facial hair or hair on body for males) and internalizing and externalizing symptoms at age 11. These were assessed using the mother-rated Strengths and Difficulties Questionnaire (SDQ) [23]. The SDQ is a valid and reliable tool for measuring such symptoms in children [24]. It consists of 20 “difficulties” items related to behavior (in the past 6 months), with each item scored on a 3-point scale (0 = “not true,” 1 = “somewhat true,” and 2 = “certainly true”). Items can be summed to form four scales (emotional, conduct, hyperactivity, and peer problems) or two (internalizing symptoms, the sum of the scores on the emotional and peer problems items, and externalizing symptoms, the sum of the scores on the conduct and hyperactivity problems items), which we used for this analysis [25]. We also took into account an objective measure of excessive exercise collected via accelerometers. In MCS, a subsample of cohort members who participated in the age 14 sweep were asked to wear accelerometer devices for two specified full days: one during the week and the other at the weekend. The accelerometer data of MCS at age 14 express their output in “Euclidean Norm Minus One” (ENMO). This metric (ENMO) is the calculation of the average magnitude of dynamic acceleration, that is, the vector magnitude of acceleration corrected for gravity, and separates movement and gravity components in the acceleration signal. In our study, we considered the upper decile of the mean acceleration (ENMO) distribution for the week day and the weekend day to indicate moderate to vigorous physical activity.

### Statistical analysis

All analyses were performed in STATA 16.0 [26]. In all models, the MCS sampling stratum was controlled to account for the disproportionate stratification of the MCS survey design. In order to identify different ED symptom groups, we applied latent class analysis (LCA). We compared models from one to three classes. The best-fitting class solution was chosen based on the following model fit statistics: (1) The Bayesian information criterion (BIC); (2) the Akaike information criterion (AIC), and (3) the entropy of each model. Lower BIC and AIC values indicate better fit to the data. Entropy ranges from 0 to 1, with higher values indicating that the latent classes are clearly distinguishable. Maximum Likelihood with Missing Values (MLMV) was used to deal with missing data. MLMV aims to retrieve as much information as possible from observations containing missing values. We calculated the change in CGT performance between age 11 and age 14 by subtracting CGT scores at age 11 from CGT scores at age 14. We then fitted two different sets of logistic regression models in order to explore the association between the change in CGT scores between age 11 and age 14 (adjusting for CGT scores at age 11) and ED risk groups. Missingness ranged between 0.1 (ethnicity) and 28.7% (risk adjustment at age 11), and, to handle it, we used multiple imputations by chained equations (20 imputed datasets) [27].

## Results

Table S2 in the supplementary material shows the fit indices of the competing LCA models. Starting with the one-class model, stepwise additions of classes resulted in lower BIC and AIC values, suggesting a better model fit for higher order class solutions. However, the three-class model showed a lower entropy value compared to the two-class solution. Therefore, we considered the two-class solution optimal. The latent class marginal means, which represent the probability of responding positively to each question investigating ED symptoms, are shown in Table S3. We found that it was more likely that those belonging to class one 1: (a) did not have a perception of themselves as too overweight; (b) were not intending to try to lose weight; (c) were not actively restricting their nutritional intake to lose weight; (d) were not exercising to influence their body weight, and (e) were more likely to have a weight that was not in the UK90 under or overweight ranges. On the contrary, it was more likely that those belonging to class one 2: (a) had a perception of themselves as too overweight; (b) were intending to try to lose weight; (c) were restricting their nutritional intake to lose weight; (d) were exercising to influence their body weight; and (e) had an average, or above average (overweight) weight, according to the UK90. Based on these results we defined class one as being at “low risk” for ED symptoms and class two as the group “at higher risk” for ED symptoms.

Table 1 shows the descriptive statistics of the study variables in the analytic sample and the differences between the two classes. As can be seen, a higher number of white children and a lower number of Pakistani and Bangladeshi children comprised the group at lower risk for ED symptoms. In terms of CGT patterns, the group at higher risk for ED symptoms showed lower scores in risk-taking, quality of decision-making, and risk adjustment at age 11. The same individuals also showed higher scores in deliberation time and had higher levels of internalizing symptoms. Moreover, they were more likely to be in a family below the poverty line. At age 14 the higher risk group for ED symptoms still had lower scores in risk-taking, quality of decision-making, and risk adjustment but also showed higher scores in delay aversion compared to the lower risk group. Exploring the change in CGT scores between age 11 and age 14 we found that, across time, the group at higher risk for ED symptoms had a higher reduction in deliberation time scores and a lower increase in risk adjustment scores. Tables S4 and S5 in the supplementary material show the descriptive statistics of the study variables in males and females respectively. As can be seen, in males the higher risk for ED symptoms group had higher scores in risk-taking, delay aversion, internalizing and externalizing symptoms and lower scores in risk adjustment, quality of decision-making at age 11 and IQ. At age 14, they kept lower scores in risk adjustment and quality of decision-making and higher scores in delay aversion. In females, the higher risk for ED symptoms group had lower scores in quality of decision-making at ages 11 and 14 and lower scores in risk adjustment at age 14 only. They also showed higher internalizing and externalizing symptoms at age 11.

**Table 1.** Distribution between classes of the study variables in the analytic sample (*n* = 11,303) (unweighted).

	* Low risk for ED symptoms (n = 5,640) *		* Higher risk for ED symptoms (5,663) *		* Test *
	*N*	*M* (SD)	*N*	*M* (SD)	*F*
	***Continuous variables***				
Risk taking, age 11	5,157	0.53 (0.16)	5,144	0.52 (0.16)	**8.31** ^**^
Quality of decision making, age 11	5,158	0.81 (0.16)	5,144	0.79 (0.17)	**17.62** ^**^
Deliberation time, age 11	5,158	3297.08 (1358.39)	5,144	3362.80 (1296.98)	**6.31** ^*^
Risk adjustment, age 11	4,090	1.05 (0.82)	3,962	1.00 (0.81)	**7.20** ^**^
Delay-aversion, age 11	4,609	0.33 (0.19)	4,592	0.33 (0.20)	1.78
Risk taking, age 14	5,321	0.52 (0.14)	5,366	0.51 (0.14)	**10.36** ^**^
Quality of decision making, age 14	5,321	0.88 (012)	5,367	0.87 (0.13)	**16.77** ^**^
Deliberation time, age 14	5,321	2343.08 (994.28)	5,367	2334.92 (897.56)	0.20
Risk adjustment, age 14	4,711	1.24 (0.86)	4,642	1.15 (0.80)	**27.23** ^**^
Delay-aversion, age 14	4,956	0.29 (0.17)	4,987	0.30 (0.18)	**13.20** ^**^
Change in risk taking	4,838	−0.01 (0.18)	4,847	0.00 (0.18)	0.20
Change in quality of decision making	4,839	0.07 (0.17)	4,848	0.08 (0.17)	0.90
Change in deliberation time	4,839	−960.73 (1426.14)	4,848	−1039.56 (1312.36)	**8.01** ^**^
Change in risk adjustment	3,502	0.25 (1.06)	3,330	0.20 (1.03)	**5.16** ^*^
Change in delay-aversion	4,049	−0.03 (0.23)	4,047	−0.03 (0.24)	1.44
IQ, age 5	5,250	101.3 (14.91)	5,255	100.98 (14.72)	1.35
Internalizing symptoms, age 11	5,234	2.98 (3.00)	5,211	3.36 (3.22)	**39.20** ^**^
Externalizing symptoms, age 11	5,236	4.30 (3.51)	5,203	4.42 (3.51)	2.94
	***Categorical variables***				
	***N***	**%**	***N***	**%**	**Chi** ^**2**^
*Pubertal status (female)*					
Yes	2,799	58.8	3,563	75.16	**284.98** ^**^
No	1,955	41.2	1,177	24.84	
*Pubertal status (male)*					
Yes	2,047	42.65	2,639	55.72	**162.85** ^**^
No	2,752	57.35	2,097	44,28	
Gender—female	2,239	39.69	3,432	60.60	**493.98** ^**^
Gender—male	3,401	60.31	2,231	39.40	
Below poverty line	1,275	22.60	1,436	25.35	**11.73** ^**^
Above poverty line	4,365	77.40	4,227	74.65	
*Ethnicity*					
White	4,671	82.95	4,579	80.90	**8.01** ^**^
Mixed	155	2.71	168	2.96	0.47
Indian	145	2.57	163	2.87	0.98
Pakistani and Bangladeshi	403	7.15	464	8.19	**4.31** ^*^
Black or Black British	171	3.03	192	3.39	1.14
Other Ethnic group	86	1.52	94	1.66	0.32
Upper decile physical activity	428	17.88	305	13.15	**20.03** ^**^
Lower deciles physical activity	1,965	82.12	2,013	86.85	

* *p* < 0.05.

** *p* < 0.01.

In Table 2 we report the correlation coefficients of the CGT variables at both time-points for the full analytic sample. As shown, correlations were generally low to moderate but, as expected, higher within-domain. That is CGT scores at age 11 correlated with CGT scores at age 14 within domain. Across domains, correlations were largest, consistently across time-points, for risk adjustment and risk-taking (−0.36 for age 11; −0.30 for age 14).

**Table 2.** Pearson’s correlations of the CGT variables at both time-points in the analytic sample (*n* = 11,303).

	Risk taking	Quality of decision making	Deliberation time	Risk adjustment	Delay aversion	Risk taking	Quality of decision making	Deliberation time	Risk adjustment	Delay aversion
	Age 11	Age 11	Age 11	Age 11	Age 11	Age 14	Age 14	Age 14	Age 14	Age 14
Risk taking	1									
Age 11										
Quality of decision making	0.09^**^	1								
Age 11										
Deliberation time	−0.05^**^	−0.21^**^	1							
Age 11										
Risk adjustment	−0.36^**^	0.08^**^	−0.05^**^	1						
Age 11										
Delay aversion	0.12^**^	−0.12^**^	−0.11^**^	−0.17^**^	1					
Age 11										
Risk taking	0.32^**^	0.02^*^	−0.03^**^	−0.07^**^	0.03^**^	1				
Age 14										
Quality of decision making	−0.00	0.33^**^	−0.08^**^	0.09^**^	−0.10^**^	0.07^**^	1			
Age 14										
Deliberation time	0.01	−0.18^**^	0.30^**^	−0.06^**^	0.03^**^	−0.05^**^	−0.36^**^	1		
Age 14										
Risk adjustment	−0.04^**^	0.18^**^	−0.08^**^	0.22^**^	−0.06^**^	−0.30^**^	0.23^**^	−0.15^**^	1	
Age 14										
Delay aversion	0.08^**^	−0.08^**^	0.00	−0.08^**^	0.16^**^	0.19^**^	−0.11^**^	−0.06^**^	−0.25^**^	1
Age 14										

* *p* < 0.05.

** *p* < 0.01.

We display the results (on both complete and imputed cases) of our logistic regression models in Table 3. As can be seen, our unadjusted model (Model 1) on imputed data showed that 1-unit increases in risk-taking and risk adjustment at age 11 were associated, respectively with 47 and 11% lower probability of being at higher risk for ED symptoms at age 14 (risk-taking *b* = −0.62, OR = 0.53, *p* < 0.01; risk adjustment *b* = −0.11, OR = 0.89, *p* < 0.05). We obtained the same results in the imputed and complete cases analysis. In our fully adjusted model (Model 2) after imputation we found that for a 1-unit increase in risk-taking score at age 11 the odds of being at higher risk for ED symptoms at age 14 increased by 2.7 times (*b* = 1.00, OR = 2.73, *p* < 0.01). In contrast, a 1-unit increase in quality of decision-making score at age 11 was associated with a 62% lower probability of being at higher risk for ED symptoms at age 14 (*b* = −0.94, OR = 0.38, *p* < 0.05). Importantly, the change in risk-taking scores between age 11 and age 14 was also associated with the odds of being in the higher risk groups for ED symptoms. A smaller reduction in risk-taking over time was associated with a higher probability of being at higher risk for ED symptoms at age 14 (*b* = 0.62, OR = 1.87, *p* < 0.05).

**Table 3:** Logistic regression models showing the probability of belonging to the higher vs low risk for ED symptoms.

	Imputed cases (*n* = 11,303)			Complete cases		
	*Model 1*			*Model 1 (n = 5,768)*		
	*b*	SE	OR	*b*	SE	OR
Risk taking	**−0.62** ^*^	0.28	0.53	**−0.62** ^*^	0.28	0.53
Age 11						
Quality of decision making	−0.54	0.39	0.58	−0.54	0.39	0.58
Age 11						
Deliberation time	−0.00	0.00	0.99	−0.00	0.00	0.99
Age 11						
Risk adjustment	**−0.11** ^*^	0.05	0.89	**−0.11** ^*^	0.05	0.89
Age 11						
Delay-aversion	0.25	0.29	1.28	0.25	0.29	1.28
Age 11						
Change in risk taking	−0.29	0.28	0.74	−0.29	0.28	0.74
Change in quality of decision making	−0.33	0.36	0.71	−0.33	0.36	0.71
Change in deliberation time	−0.00	0.00	0.99	−0.00	0.00	0.99
Change in risk adjustment	−0.08	0.04	0.91	−0.08	0.04	0.91
Change in delay-aversion	0.01	0.21	1.01	0.01	0.21	1.01
	*Model 2*			*Model 2 (n = 2,139)*		
Risk taking	**1.00** ^**^	0.32	2.73	**1.17** ^*^	0.53	3.22
Age 11						
Quality of decision making	**−0.94** ^*^	0.39	0.38	−0.09	0.66	0.91
Age 11						
Deliberation time	−0.00	0.00	0.99	0.00	0.00	1.00
Age 11						
Risk adjustment	0.05	0.05	1.05	0.01	0.09	1.01
Age 11						
Delay-aversion	0.17	0.31	1.19	0.40	0.52	1.50
Age 11						
Change in risk taking	**0.62** ^*^	0.29	1.87	0.74	0.49	2.09
Change in quality of decision making	**−**0.58	0.37	0.55	−0.19	0.61	0.82
Change in deliberation time	−0.00	0.00	0.99	−0.00	0.00	0.99
Change in risk adjustment	0.02	0.05	1.02	0.02	0.07	1.02
Change in delay-aversion	−0.14	0.23	0.86	0.01	0.39	1.02

Model 1 = CGT measures; Model 2 = Model 1+ gender, ethnicity, SES, IQ at age 5, pubertal status, exact age, internalizing and externalizing symptoms at age 11 and accelerometer data at age 14.

* *p* < 0.05.

** *p* < 0.01.

## Discussion

To our knowledge, this is the first general-population study to investigate the role of change in decision-making skills (risk-taking, quality of decision-making, risk adjustment, deliberation time, delay aversion) over time in adolescence in ED symptomatology. We explored this across two time-points in adolescence (ages 11 and 14 years) and found that those at higher risk of ED symptoms at age 14 years had at the previous time-point (age 11 years) higher scores on risk-taking and lower scores on quality of decision-making. The association was robust to adjustment for decision-making skills at age 14 as well as confounders. Importantly, a smaller reduction in risk-taking over time (between ages 11 and 14) was associated with a higher risk of developing ED symptoms at age 14.

Our LCA identified two distinct groups. Individuals belonging to group 1 were at low risk of developing ED symptoms as they showed: lower risk of perceiving their body as overweight; less intention to lose weight; less nutritional restriction; less use of exercise with the purpose of influencing their body weight; and lower risk of being under or overweight according to the UK90 reference cut-offs. On the contrary, individuals belonging to group 2 were at higher risk of developing ED symptoms as they showed: higher risk of perceiving their body as overweight; more intention to lose weight; more nutritional restriction; more use of exercise with the purpose of influencing their body weight; and higher risk of being overweight according to the UK90 reference cut-off. The group at higher risk for ED symptoms showed significantly lower scores on risk-taking, quality of decision-making, and risk adjustment at age 11 compared to the group at low risk for ED symptoms. We observed the same results in the two groups at age 14. Moreover, at age 14 the group at higher risk for ED symptoms showed higher scores on delay aversion. Deliberation time was slower for that group at age 11 but the two groups did not differ in the age 14 scores. Focusing on the change over time in the CGT scores, we found that at age 14, compared to age 11, both groups appeared to show less risk-taking, deliberation time, and delay aversion but the greater quality of decision-making and risk adjustment.

Overall, the *direction* of these changes suggests that both groups appeared to follow, broadly, the same developmental trajectories of decision-making skills. However, in our descriptive “unadjusted” analysis, we also found that there were some significant differences in the *amount* of change for the two groups. The higher risk group displayed a greater reduction in deliberation time and less improvement in risk adjustment, compared to the group at low risk for ED symptoms. The finding with respect to risk adjustment can be taken as evidence for the much-discussed inflexibility in reward processing shown by those at risk of ED symptoms. The finding with respect to the reduction in deliberation time must be considered alongside the two groups’ absolute levels of deliberation time in the two time-points. The higher risk group did not differ in absolute terms from their low-risk counterparts at age 14. They merely showed much slower times at age 11. Together, these findings suggest that developmental lags in response (or processing) time may indicate the risk of ED symptoms in the general adolescent population. This is important to consider alongside the null findings with respect to group differences in IQ in our sample. Overall, we observe a small improvement in decision-making in the “normative” group, in line with the evidence on the average trajectory of decision-making across adolescence described in previous studies [10]. Our findings are also in line with other studies that found impaired decision-making abilities in adolescents with EDs [28,29].

In our final regression model controlling for all covariates, the association between risk-taking at age 11 and the risk for ED symptoms at age 14 was reversed. That is, risk-taking at age 11 was related positively to risk for ED symptoms at age 14 after adjustment for confounders. This phenomenon is sometimes known as the Lord’s paradox [30] and is relevant to situations where some of the covariates lie on a causal pathway. In our case, the risk for developing ED symptoms was higher when risk-taking was higher after adjusting for gender. These findings are in line with previous studies about gender differences [18] in decision-making and EDs [31]. In addition, we found that better quality of decision-making at age 11 was associated with a lower likelihood of being at risk for ED symptoms at age 14, and, as discussed earlier, that less reduction over time in risk-taking was associated with the risk of developing ED symptoms. Less reduction in risk-taking scores across this period may be taken to suggest that trait-like reward sensitivity early in adolescence may be a putative risk factor for later EDs. This, in turn, would be in line with findings from neurobiological studies about a dysregulated reward circuitry function in EDs [32,33].

Our findings come with some limitations. First, given the multidisciplinary nature of the MCS, we did not have a clinical interview for EDs available. However, we used a broad classification system to detect ED symptomatology which may be more appropriate with a general population sample. For instance, the residual “eating disorder not otherwise specified” category, which describes the most heterogeneous ED category, has been consistently found as the most common ED diagnosis in clinical samples. These results highlight the importance of using a broader approach in considering ED symptomatology especially when investigating a young community sample. Second, ED symptoms in MCS were measured for the first time at age 14 so we could not control for them at age 11, when we first measured decision-making. Third, we found significant, albeit very small, differences in CGT scores between the two groups, which suggests that CGT may have limited value in clinical practice. Nevertheless, it is important to keep in mind that both our groups come from a general population cohort. Thus, one should not expect to find the same differences typically shown between clinical and healthy control groups. Fourth, as we used the recommended cut-offs for underweight and overweight from the UK90, the second centile likely underestimates underweight and is not direct equivalent to the 85th centile for overweight. This could explain why we find a relatively small number of individuals in the cohort with significant underweight. In future work, we could consider other cut-offs, such as weight for height percentiles based on World Health Organization Growth Charts. Finally, there are the limitations of the CGT. Risk-taking conflates the seeking of reward and the avoidance of punishment. Reduced betting, even when the odds of winning are high, might occur because participants are less motivated by reward or because they want to avoid loss (punishment), and this cannot be disentangled in this task.

## Conclusions

This study sheds light on the role of decision-making skills in ED symptomatology in adolescence. If replicated in a clinical sample our results might help to structure new interventions targeting modifiable risk factors for the development of EDs, thus helping young individuals to achieve better outcomes. We filled an important gap in the literature by showing how the development of decision-making under risky conditions across early and middle adolescence may be related to the risk for ED psychopathology in middle adolescence. Future studies should explore our findings in older populations (i.e., late adolescence/young adulthood) as well.

## Data Availability

The data that support the findings of this study are openly available from the UK data service website (https://ukdataservice.ac.uk/).

## Supplementary material

For supplementary material accompanying this paper visit https://doi.org/10.1192/j.eurpsy.2020.92. click here to view supplementary material

## References

[1] Smink FR, van Hoeken D, Hoek HW. Epidemiology, course, and outcome of eating disorders. Current Opin Psychiatry. 2013;26(6):543–8. 10.1097/YCO.0b013e328365a24f 24060914

[2] Schmidt U, Adan R, Böhm I, Campbell I, Dingemans A, Ehrlich S, et al. Eating disorders: the big issue. Lancet Psychiatry. 2016;3(4):313–315. 27063378 10.1016/S2215-0366(16)00081-X

[3] Steinhausen H-C. Outcome of eating disorders. Child Adolesc Psychiatr Clin N Am. 2009;18(1):225–242. 19014869 10.1016/j.chc.2008.07.013

[4] Pratt BM, Woolfenden S. Interventions for preventing eating disorders in children and adolescents. Cochrane Database Syst Rev. 2002;(2):CD002891. 10.1002/14651858.CD002891 PMC6999856 12076457

[5] Rosen DS. Identification and management of eating disorders in children and adolescents. Pediatrics. 2010;126(6):1240–1253. 21115584 10.1542/peds.2010-2821

[6] Keel PK, Brown TA. Update on course and outcome in eating disorders. Int J Eating Disorders. 2010;43(3):195–204. 10.1002/eat.20810 20186717

[7] Kessler RC, Berglund PA, Chiu WT, Deitz AC, Hudson JI, Shahly V, et al. The prevalence and correlates of binge eating disorder in the World Health Organization World Mental Health Surveys. Biol Psychiatry. 2013;73(9):904–914. 23290497 10.1016/j.biopsych.2012.11.020 PMC3628997

[8] Harrison A, O'Brien N, Lopez C, Treasure J. Sensitivity to reward and punishment in eating disorders. Psychiatry Res. 2010;177(1–2):1–11. 20381877 10.1016/j.psychres.2009.06.010

[9] Harrison A, Sternheim L, O'Hara C, Oldershaw A, Schmidt U. Do reward and punishment sensitivity change after treatment for anorexia nervosa? Personality Indiv Diff. 2016;96:40–46.

[10] Smith DG, Xiao L, Bechara A. Decision making in children and adolescents: impaired Iowa gambling task performance in early adolescence. Dev Psychol. 2012;48(4):1180. 22081879 10.1037/a0026342

[11] Kaye W. Neurobiology of anorexia and bulimia nervosa. Physiol Behav. 2008;94(1):121–135. 18164737 10.1016/j.physbeh.2007.11.037 PMC2601682

[12] Matton A, Goossens L, Braet C, Vervaet M. Punishment and reward sensitivity: are naturally occurring clusters in these traits related to eating and weight problems in adolescents? Eur Eating Disorders Rev. 2013;21(3):184–194. 10.1002/erv.2226 23426856

[13] Treasure J, Schmidt U. The cognitive-interpersonal maintenance model of anorexia nervosa revisited: a summary of the evidence for cognitive, socio-emotional and interpersonal predisposing and perpetuating factors. J Eating Disorders. 2013;1(1):13. 10.1186/2050-2974-1-13 PMC4081714 24999394

[14] Giedd JN, Blumenthal J, Jeffries NO, Castellanos FX, Liu H, Zijdenbos A, et al. Brain development during childhood and adolescence: a longitudinal MRI study. Nat Neurosci. 1999;2(10):861–863. 10491603 10.1038/13158

[15] Hooper CJ, Luciana M, Conklin HM, Yarger RS. Adolescents’ performance on the Iowa gambling task: implications for the development of decision making and ventromedial prefrontal cortex. Dev Psychol. 2004;40(6):1148. 15535763 10.1037/0012-1649.40.6.1148

[16] Deakin J, Aitken M, Robbins T, Sahakian BJ. Risk taking during decision-making in normal volunteers changes with age. J Int Neuropsychol Soc. 2004;10(4):590, 8. 15327737 10.1017/S1355617704104104

[17] Rogers RD, Owen AM, Middleton HC, Williams EJ, Pickard JD, Sahakian BJ, et al. Choosing between small, likely rewards and large, unlikely rewards activates inferior and orbital prefrontal cortex. J Neurosci. 1999;19(20):9029–9038. 10516320 10.1523/JNEUROSCI.19-20-09029.1999 PMC6782753

[18] Van Leijenhorst L, Westenberg PM, Crone EA. A developmental study of risky decisions on the cake gambling task: age and gender analyses of probability estimation and reward evaluation. Dev Neuropsychol. 2008;33(2):179–196. 18443976 10.1080/87565640701884287

[19] Plewis I, Calderwood L, Hawkes D, Hughes G, Joshi H. Millennium Cohort Study: technical report on sampling. London: Centre for Longitudinal Studies, 2007.

[20] Robbins TW, James M, Owen AM, Sahakian BJ, Lawrence AD, McInnes L, et al. A study of performance on tests from the CANTAB battery sensitive to frontal lobe dysfunction in a large sample of normal volunteers: implications for theories of executive functioning and cognitive aging. J Int Neuropsychol Soc. 1998;4(5):474–490. 9745237 10.1017/s1355617798455073

[21] Cole TJ, Freeman JV, Preece MA. Body mass index reference curves for the UK, 1990 Arch Dis Childhood. 1995;73(1):25–9. 10.1136/adc.73.1.25 PMC1511150 7639544

[22] Elliot CD, Smith P, McCulloch K. British ability scales II. Windsor: NFER-Nelson, 1996.

[23] Goodman R. The Strengths and Difficulties Questionnaire: a research note. J Child Psychol Psychiatry. 1997;38(5):581–586. 9255702 10.1111/j.1469-7610.1997.tb01545.x

[24] Goodman R. Psychometric properties of the Strengths and Difficulties Questionnaire. J Am Acad Child Adolesc Psychiatry. 2001;40(11):1337–1345. 11699809 10.1097/00004583-200111000-00015

[25] Goodman A, Lamping DL, Ploubidis GB. When to use broader internalising and externalising subscales instead of the hypothesised five subscales on the Strengths and Difficulties Questionnaire (SDQ): data from British parents, teachers and children. J Abnorm Child Psych. 2010;38(8):1179–1191. 10.1007/s10802-010-9434-x 20623175

[26] StataCorp. 2017 Stata Statistical Software: Release 15 College Station, TX: StataCorp LLC.

[27] Royston P, White IR. Multiple imputation by chained equations (MICE): implementation in Stata. J Stat Softw. 2011;45(4):1–20.

[28] Kittel R, Schmidt R, Hilbert A. Executive functions in adolescents with binge‐eating disorder and obesity. Int J Eating Disorders. 2017;50(8):933–941. 10.1002/eat.22714 28407284

[29] Reiter AM, Heinze H-J, Schlagenhauf F, Deserno L. Impaired flexible reward-based decision-making in binge eating disorder: evidence from computational modeling and functional neuroimaging. Neuropsychopharmacology. 2017;42(3):628–637. 27301429 10.1038/npp.2016.95 PMC5240187

[30] Tu Y-K, Gunnell D, GMS . Simpson’s paradox, Lord’s paradox, and suppression effects are the same phenomenon—the reversal paradox. Emerg Themes Epidemiol. 2008;5(1):2. 18211676 10.1186/1742-7622-5-2 PMC2254615

[31] Harvey JA, Robinson JD. Eating disorders in men: current considerations. J Clin Psychol Med Settings. 2003;10(4):297–306.

[32] Dichter GS, Damiano CA, Allen JA. Reward circuitry dysfunction in psychiatric and neurodevelopmental disorders and genetic syndromes: animal models and clinical findings. J Neurodev Disord. 2012;4(1):19. 22958744 10.1186/1866-1955-4-19 PMC3464940

[33] Guillaume S, Gorwood P, Jollant F, Van den Eynde F, Courtet P, Richard-Devantoy S. Impaired decision-making in symptomatic anorexia and bulimia nervosa patients: a meta-analysis. Psychol Med. 2015;45(16):3377–3391. 26497047 10.1017/S003329171500152X

