# Supplementary material for: Change in decision-making skills and risk for eating disorders in adolescence: A population-based study
Source: Eur Psychiatry. 2020 Oct 13;63(1):e93. doi: 10.1192/j.eurpsy.2020.92 (PMC7681158; doi:10.1192/j.eurpsy.2020.92)
Supplement: Supplementary file 1 [file S0924933820000929sup001.docx]

| **Table S1. Outcome Measures from the Cambridge Gambling Task** | |
| --- | --- |
| **Outcome Variable** | **Description** |
| **Risk-taking** | The mean proportion of the current points total that the participant chooses to gamble on trials when they selected the most probable colour. |
| **Quality of decision-making** | The mean proportion of trials where the most probable colour was selected. |
| **Deliberation-time** | Time (in milliseconds) of how long it takes the participant to decide which colour of box is hiding the token. |
| **Risk-adjustment** | The extent to which, on trials where a larger proportion of boxes are a certain colour, participants bet a higher proportion of their points. Higher risk adjustment scores represent a higher proportion of points bet as ratio increases |
| **Delay-aversion** | The time a participant is prepared to wait in order to place a higher or lower bet. It is the difference between the risk-taking score in the descend condition and that in the ascend condition. To the degree that the participant has difficulty in withholding a response over a delay, they will tend to select an amount to bet which occurs early in the sequence; i.e., a large bet in the descend condition, and a small bet in the ascend condition |

| **Table S2. Goodness-of-fit statistics** **of the competing LCA models** | | | |
| --- | --- | --- | --- |
|  | **One-class solution** | **Two-class solution** | **Three-class solution** |
| Entropy |  | 0.7831010 | 0.6317767 |
| AIC | 64912.40 | 54762.268 | 54312.289 |
| BIC | 64956.39 | 54857.595 | 54458.945 |

| **Table S3.** **Latent class marginal means** | | | |
| --- | --- | --- | --- |
| **Variable Name** | **Margin** | **SD** | **95% CI** |
| **Class 1 (low risk)** | | | |
| Body dissatisfaction | 0.00 | 0.00 | 0.00 **−** 0.00 |
| Dieting behaviours | 0.04 | 0.00 | 0.03 − 0.05 |
| Dietary restraint | 0.08 | 0.00 | 0.07 − 0.09 |
| Excessive exercise | 0.28 | 0.00 | 0.26 − 0.29 |
| Significant Under/Over weight cut-off | | | |
| 0 (Normal) | 0.84 | 0.00 | 0.82 − 0.85 |
| 1 (Under) | 0.02 | 0.00 | 0.02 − 0.03 |
| 2 (Over) | 0.12 | 0.00 | 0.11 − 0.14 |
| **Class 2 (higher risk)** | | | |
| Body dissatisfaction | 0.08 | 0.00 | 0.08 − 0.09 |
| Dieting behaviours | 0.79 | 0.00 | 0.78 − 0.81 |
| Dietary restraint | 0.80 | 0.00 | 0.79 − 0.81 |
| Excessive exercise | 0.92 | 0.00 | 0.91 − 0.93 |
| Significant Under/Over weight cut-off | | | |
| 0 (Normal) | 0.43 | 0.00 | 0.41 − 0.44 |
| 1 (Under) | 0.00 | 0.00 | 0.00 − 0.00 |
| 2 (Over) | 0.56 | 0.00 | 0.55 − 0.58 |

| **Table S4.** **Distribution between classes of the study variables in males only (n=5,632) (unweighted)** | | | | | |
| --- | --- | --- | --- | --- | --- |
|  |  | | | | |
|  | *Class 1 (low risk) (n= 3,401)* | | *Class 2 (higher risk) (n=2,231)* | | *Test* |
|  | **N** | **M(SD)** | **N** | **M(SD)** | **F** |
|  | ***Continuous variables*** | | | | |
| Risk taking,  age 11 | 3,094 | 0.56 (0.15) | 2,011 | 0.58 (0.15) | **7.33^**^** |
| Quality of decision making,  age 11 | 3,095 | 0.81 (0.16) | 2,011 | 0.79 (0.17) | **18.53**^**^ |
| Deliberation time,  age 11 | 3,095 | 3240.90 (1316.37) | 2,011 | 3300.13 (1220.46) | 2.61 |
| Risk adjustment,  age 11 | 2,502 | 1.05 (0.80) | 1,551 | 0.98 (0.78) | **7.46**^**^ |
| Delay-aversion,  age 11 | 2,868 | 0.33 (0.18) | 1,877 | 0.35 (0.18) | **6.78**^**^ |
| Risk taking,  age 14 | 3,195 | 0.55 (0.14) | 2,105 | 0.55 (0.14) | 2.79 |
| Quality of decision making,  age 14 | 3,195 | 0.88 (012) | 2,106 | 0.87 (0.13) | **13.49^**^** |
| Deliberation time,  age 14 | 3,195 | 2358.55 (984.88) | 2,106 | 2370.37 (945.13) | 0.19 |
| Risk adjustment,  age 14 | 2,884 | 1.29 (0.87) | 1,872 | 1.22 (0.80) | **8.30^**^** |
| Delay-aversion,  age 14 | 3,023 | 0.29 (0.16) | 2,000 | 0.31 (0.16) | **13.92^**^** |
| Change in Risk taking | 2,888 | -0.01 (0.17) | 1,885 | -0.02 (0.17) | 0.48 |
| Change in Quality of decision making | 2,889 | 0.07 (0.16) | 1,886 | 0.08 (0.17) | 2.69 |
| Change in Deliberation time | 2,889 | -960.73 (1426.14) | 1,886 | -1039.56 (1312.36) | 2.38 |
| Change in Risk adjustment | 2,158 | 0.30 (1.05) | 1,331 | 0.29 (0.99) | 0.16 |
| Change in Delay-aversion | 2,357 | -0.04 (0.22) | 1,677 | -0.04 (0.22) | 0.93 |
| IQ,  age 5 | 3,163 | 100.6 (15.11) | 2,049 | 99.32 (15.38) | **10.05^**^** |
| Internalising symptoms,  age 11 | 3,143 | 2.99 (3.05) | 2,043 | 3.45 (3.27) | **26.90**^**^ |
| Externalising symptoms,  age 11 | 3,146 | 4.79 (3.68) | 2,041 | 5.15 (3.75) | **12.10^**^** |
|  | ***Categorical variables*** | | | | |
|  | **N** | **%** | **N** | **%** | **Chi^2^** |
| *Pubertal status (male)* |  | | | | |
| Yes | 1,048 | 30.81 | 749 | 33.57 | **7.83^**^** |
| No | 1,849 | 69.19 | 1,114 | 66.43 |  |
| Below poverty line | 754 | 22.60 | 569 | 25.35 | **8.33**^**^ |
| Above poverty line | 2,647 | 77.40 | 1,662 | 66.43 |  |
| **Ethnicity** | | | | | |
| White | 2,841 | 82.95 | 1,780 | 80.90 | **13.86^**^** |
| Mixed | 93 | 2.71 | 64 | 2.96 | 0.08 |
| Indian | 78 | 2.57 | 84 | 2.87 | **10.37^**^** |
| Pakistani and Bangladeshi | 220 | 7.15 | 189 | 8.19 | **7.92^*^** |
| Black or Black British | 108 | 3.03 | 69 | 3.39 | 0.03 |
| Other Ethnic group | 54 | 1.52 | 44 | 1.66 | 1.14 |
| Upper decile physical activity | 324 | 23.09 | 197 | 22.15 | 0.27 |
| Lower deciles physical activity | 1,079 | 76.91 | 692 \| | 77.85 |  |
| * p<.05 **p<.01 | | | | | |

| **Table S5.** **Distribution between classes of the study variables in females only (n=5,671) (unweighted)** | | | | | |
| --- | --- | --- | --- | --- | --- |
|  |  | | | | |
|  | *Class 1 (low risk)*  *(n=2,239)* | | *Class 2 (higher risk)*  *(3,432)* | | *Test* |
|  | **N** | **M(SD)** | **N** | **M(SD)** | **F** |
|  | ***Continuous variables*** | | | | |
| Risk taking,  age 11 | 2,063 | 0.47 (0.16) | 3,133 | 0.48 (0.16) | 2.74 |
| Quality of decision making,  age 11 | 2,063 | 0.81 (0.17) | 3,133 | 0.80 (0.17) | **4.88**^*^ |
| Deliberation time,  age 11 | 2,063 | 3381.36 (1415.25) | 3,133 | 3403.02 (1342.46) | 0.31 |
| Risk adjustment,  age 11 | 1,588 | 1.04 (0.85) | 2,411 | 1.01 (0.83) | 1.57 |
| Delay-aversion,  age 11 | 1,741 | 0.32 (0.20) | 2,715 | 0.32 (0.20) | 0.81 |
| Risk taking,  age 14 | 2,126 | 0.47 (0.14) | 3,261 | 0.48 (0.14) | 2.60 |
| Quality of decision making,  age 14 | 2,126 | 0.88 (0.13) | 3,261 | 0.87 (0.13) | **5.39^*^** |
| Deliberation time,  age 14 | 2,126 | 2319.83 (1008.03) | 3,261 | 2312.02 (864.82) | 0.09 |
| Risk adjustment,  age 14 | 1,827 | 1.17 (0.82) | 2,770 | 1.11 (0.80) | **6.14^**^** |
| Delay-aversion,  age 14 | 1,933 | 0.29 (0.19) | 2,987 | 0.30 (0.19) | 2.49 |
| Change in Risk taking | 1,950 | -0.00 (0.18) | 2,962 | -0.00 (0.18) | 0.00 |
| Change in Quality of decision making | 1,950 | 0.07 (0.17) | 2,962 | 0.07 (0.17) | 0.00 |
| Change in Deliberation time | 1,950 | -1073.05 (1496.09) | 2,962 | -1099.33 (1339.07) | 0.41 |
| Change in Risk adjustment | 1,344 | 0.18 (1.06) | 1,999 | 0.14 (1.04) | 1.31 |
| Change in Delay-aversion | 1,512 | -0.01 (0.25) | 2,370 | -0.02 (0.25) | 0.22 |
| IQ,  age 5 | 2,087 | 102.27 (14.54) | 3,206 | 102.04 (14.18) | 0.31 |
| Internalising symptoms,  age 11 | 2,091 | 2.97 (2.92) | 3,168 | 3.30 (318) | **14.85**^**^ |
| Externalising symptoms,  age 11 | 2,090 | 3.57 (3.10) | 3,162 | 3.94 (3.26) | **17.33^**^** |
|  | ***Categorical variables*** | | | | |
|  | **N** | **%** | **N** | **%** | **Chi^2^** |
| *Pubertal status (female)* |  | | | | |
| Yes | 1,916 | 99.68 | 2,915 | 99.55 | 0.51 |
| No | 6 | 0.32 | 13 | 0.45 |  |
| Below poverty line | 521 | 23.26 | 867 | 25.27 | 2.91 |
| Above poverty line | 1,718 | 76.74 | 2,565 | 74.73 |  |
| **Ethnicity** | | | | | |
| White | 1,830 | 81.80 | 2,799 | 81.60 | 0.03 |
| Mixed | 62 | 2.77 | 104 | 3.03 | 0.32 |
| Indian | 67 | 2.99 | 79 | 2.30 | 2.58 |
| Pakistani and Bangladeshi | 183 | 8.18 | 275 | 8.01 | 0.04 |
| Black or Black British | 108 | 3.03 | 69 | 3.39 | 0.03 |
| Other Ethnic group | 63 | 2.81 | 123 | 3.58 | 2.52 |
| Upper decile physical activity | 104 | 10.05 | 108 | 7.55 | **6.35**^*^ |
| Lower deciles physical activity | 886 | 89.95 | 1,321 | 92.45 |  |
| * p<.05 **p<.01 | | | | | |
